# Antifungal Activities of Fluorinated Pyrazole Aldehydes on Phytopathogenic Fungi, and Their Effect on Entomopathogenic Nematodes, and Soil-Beneficial Bacteria

**DOI:** 10.3390/ijms24119335

**Published:** 2023-05-26

**Authors:** Vesna Rastija, Karolina Vrandečić, Jasenka Ćosić, Gabriella Kanižai Šarić, Ivana Majić, Dejan Agić, Domagoj Šubarić, Maja Karnaš, Drago Bešlo, Harshad Brahmbhatt, Mario Komar

**Affiliations:** 1 Faculty of Agrobiotechnical Sciences Osijek, Josip Juraj Strossmayer University of Osijek, Vladimira Preloga 1, 31000 Osijek, Croatia; karolina.vrandecic@fazos.hr (K.V.); jasenka.cosic@fazos.hr (J.Ć.); gabriella.kanizai@fazos.hr (G.K.Š.); ivana.majic@fazos.hr (I.M.); dagic@fazos.hr (D.A.); domagoj.subaric@fazos.hr (D.Š.); maja.karnas@fazos.hr (M.K.); drago.beslo@fazos.hr (D.B.); 2 Faculty of Food Technology Osijek, Josip Juraj Strossmayer University, Franje Kuhača 20, 31000 Osijek, Croatia; brahmbhathashad@hotmail.com (H.B.); mario.komar@ptfos.hr (M.K.)

**Keywords:** pyrazole aldehydes, plant protection, antifungal activity, antibacterial activity, nematicidal activity, molecular docking, toxicity

## Abstract

Fluoro-substituted pyrazoles have a wide range of biological activities, such as antibacterial, antiviral, and antifungal activities. The aim of this study was to evaluate the antifungal activities of fluorinated 4,5-dihydro-1*H*-pyrazole derivatives on four phytopathogenic fungi: *Sclerotinia sclerotiorum*, *Macrophomina phaseolina*, *Fusarium oxysporum* f. sp*. lycopersici*, and *F. culmorum*. Moreover, they were tested on two soil beneficial bacteria—*Bacillus mycoides* and *Bradyrhizobium japonicum*—as well as two entomopathogenic nematodes (EPNs)—*Heterorhabditis bacteriophora* and *Steinernema feltiae*. The molecular docking was performed on the three enzymes responsible for fungal growth, the three plant cell wall-degrading enzymes, and acetylcholinesterase (AChE). The most active compounds against fungi *S. sclerotiorum* were 2-chlorophenyl derivative (**H9**) (43.07% of inhibition) and 2,5-dimethoxyphenyl derivative (**H7**) (42.23% of inhibition), as well as **H9** against *F. culmorum* (46.75% of inhibition). Compounds were shown to be safe for beneficial soil bacteria and nematodes, except for compound **H9** on EPN *H. bacteriophora* (18.75% mortality), which also showed the strongest inhibition against AChE (79.50% of inhibition). The molecular docking study revealed that antifungal activity is possible through the inhibition of proteinase K, and nematicidal activity is possible through the inhibition of AChE. The fluorinated pyrazole aldehydes are promising components of future plant protection products that could be environmentally and toxicologically acceptable.

## 1. Introduction

Since plant pests and diseases are responsible for major economic losses in agriculture, the application of plant protection products is crucial. However, increasing resistance to pesticides, as well as their environmental and health hazards, indicate an urgent need for finding new active components for plant protection products [1]. Directive 2009/128/EC of the European Parliament and the European Council [2] established a framework to achieve sustainable use of pesticides that have the most negligible side effects on human health, non-target organisms, and environmentally and toxicologically acceptable organisms. European Union proposed the following development strategy for creating new pesticides: (1) the development of pesticides that are effective at an extremely low dosage, (2) the development of pesticides that are readily degradable and less residual in the environment, and (3) the development of selective toxic agrochemicals [3].

Fungi are among the dominant causal agents of plant diseases [4]. *Sclerotinia sclerotiorum* (Lib.) de Bary is a polyphagous, ubiquitous, and destructive plant pathogen that causes seed decay, root, and stem rot and white mold of fruits of more than 400 plant species. It is a major threat to crop production because of limited gene resources for disease resistance [5]. *Macrophomina phaseolina* (Tassi) Goid. is a polyphagous phytopathogen that causes diseases in numerous cultivated (e.g., sunflower, soybean, rapeseed, tobacco, carrot, hemp, sorghum, and cotton) and wild plant species. Diseases caused by this fungus increased in recent years due to high temperatures and low soil moisture that favor its occurrence [6]. *Fusarium* is one of the most economically important plant pathogens in the world. [7]. *Fusarium oxysporum* f. sp. *lycopersici* (Sacc.) (W.C. Snyder and H.N. Hansen) is a growing problem in tomato production, especially in field cultivation [8]. The management of tomato wilt is complex, and novel approaches are necessary. *Fusarium culmorum* (Wm.G. Sm.) Sacc. is predominant fungal pathogen affecting small grain cereals, especially in cooler production areas, all over the world [9]. Pathogen interactions could enhance each other’s aggressiveness. Thus, the aggressiveness of *F. culmorum* was related to interactions with *F. oxysporum*, *F. sambucinum* var. minus, and *Bipolaris sorokiniana* [10].

Entomopathogenic nematodes (EPNs) are beneficial organisms used in insect pest management programs. They are often either combined with plant stimulants, inorganic, and organic plant fertilizers, or evaluated using chemical pesticide compatibility Entomopathogenic nematodes. *Heterorhabditis bacteriophora* and *Steinernema feltiae* are beneficial nematodes used in insect pest management programs [11]. These soil-dwelling organisms are an excellent model to investigate the biological activity of chemical compounds and their effects on beneficial soil fauna, in addition to their comparative value to the free-living nematode *Caenorhabditis elegans*. Due to genetic tractability, experimental versatility, and evolutionary conservation, *C. elegans* serves as an important model organism to explore fundamental ecological principles and ecological interactions and make connections between genes, behavior, and the environment. Entomopathogenic nematodes and *C. elegans* are both nematodes; however, they differ in their ecological roles and sensitivity to chemicals. When evaluating the negative impact of chemicals on soil fauna, using EPNs as a test organism provides a more ecologically relevant perspective compared to using *C. elegans*. The goal of successful nematode pest control is the lethal effect on plant-parasitic nematodes (PPNs) and no effect on EPNs [12]. The actions of some active components of plant protection products are related to the inhibition of the enzyme acetylcholinesterase (AChE), which catalyzes acetylcholine hydrolysis, which is an essential neurotransmitter in the central nervous system of insects, rodents and humans [13]. Atwa et al. [14] demonstrate that differences in the survival percentages of entomopathogenic nematodes in the families *Steinernematidae* and *Heterorhabditidae* may be attributed to differences in nematodes’ AChE concentration.

Previous research [15] determined the inhibitory effect of fluorinated pyrazoles aldehydes on the pathogen bacterial populations. However, the effect of tested compounds on representatives of beneficial soil bacterial species has not been tested. The following organisms are included as model organisms: *Bacillus mycoides* (gram-positive) and *Bradyrhizobium japonicum* (gram-negative), which are ubiquitous and widespread in the soil and show PGPR (plant growth promoting) activity with an important role in nitrogen cycling and maintenance of soil fertility [16,17].

**Scheme 1:**
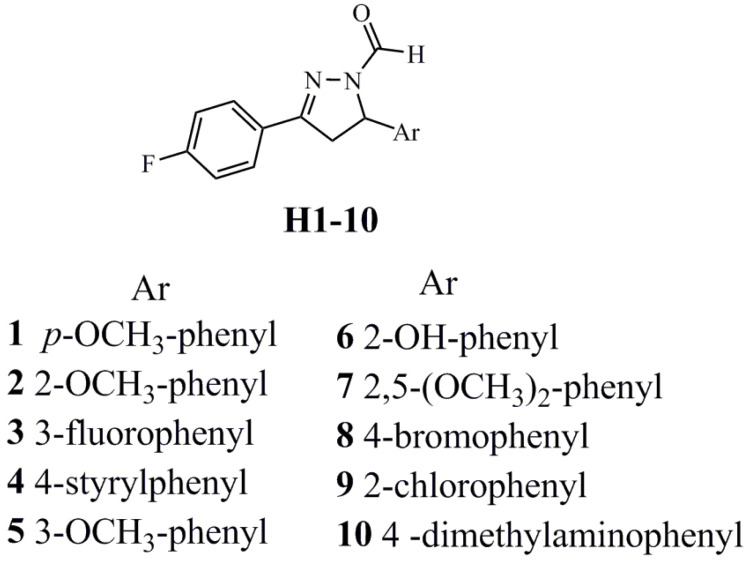
Structures of analysed antifungal agents, fluorinated pyrazole aldehydes (**H1**–**H10**). Their synthesis and characterization were reported previously [15].

Pyrazoles are heterocyclic organic compounds with a 5-membered ring structure composed of three carbon atoms and two nitrogen atoms in adjacent positions [18]. Recently, we synthesized a series of fluorinated 4,5-dihydro-1*H*-pyrazole derivatives in the reaction of corresponding acetophenone and aldehydes, followed by the second step synthesis from chalcone, hydrazine hydrate, and formic acid (Scheme 1) [15]. Several biological activities were reported for pyrazoline derivatives, including antibacterial [19,20,21], antiviral [22,23], and antifungal activities [24,25,26]. A series of cycloadducts–pyrazoles exhibited significant fungicidal activities against the necrotrophic plant pathogen *Corynespora cassiicola* [24]. Isoxazolol pyrazole carboxylate exhibited strong antifungal activity against *Rhizoctonia solani* [25]. The novel pyrazole–thiazole carboxamides showed excellent in vitro activities against *Rhizoctonia cerealis*; these activities were superior to those of the commercial fungicide thifluzamide [26]. Fluoro-substituted phenylpyrazole exhibited a wide range of activities related to plant protection, such as antibacterial, insecticidal, and acaricidal activities [27,28,29,30]. The introduction of fluorine atoms into organic molecules affects their steric, lipophilic, and electronic parameters, improving the environmental and inhibitory effects of pesticides [31]. Difluoromethyl-substituted pyrazoles are fungicides, such as bixafen, isopyrazam, sedaxane, fluxapyroxad, and benzovindiflupyr [32]. Thus, benzovindiflupyr, which was developed by Syngenta, is a pyrazole carboxamide fungicide acting as a succinate dehydrogenase inhibitor. However, pyraziflumid, which contains two different fluorinated moieties, shows excellent biological activity against ascomycete and basidiomycete fungi [33]. Moreover, new fluorinated Tebufenpyrad analogs showed stronger acaricidal activity than those commercial compounds [34].

Computer-aided molecular design (CAMD) is generally accepted and extensively applied in the ecotoxicological modeling and design of agrochemicals for its high efficiency in the design of new compounds, saving both time and economic costs in large-scale experimental synthesis and biological tests. CAMD is also an important tool for the prediction of toxicity, which can greatly reduce the risk of environmental contamination and prevent potential hazards caused by the excessive use of agrochemicals. Moreover, it allows the design of compounds that are more biodegradable and less toxic [35].

Molecular docking is a valuable tool for drug discovery, and was also recently used in the discovery of novel plant protection agents. This technique reveals the mechanism of action of a potential pesticide at the atomic level. It allows insight into the interactions between a small molecule (ligand) and the binding site of target proteins (receptors) related to biological activity. Molecular docking involves the prediction of the ligand orientation within the binding site of the protein, as well as the evaluation of binding affinity between the receptor and the ligand via the scoring function (binding energy) [36,37]. For the research of antifungal agents’ mechanisms of action, possible target in molecular docking are enzymes responsible for fungal growth or progress into the host organism. One of the enzymes required for the fungal growth is cytochrome P450 sterol 14*α*-demethylase (CYP51, syn. ERG11). The CYP51 enzymes of fungal pathogens use eburicol or lanosterol as substrates to produce ergosterol [38]. Many field studies showed that sterol demethylation inhibitor (DMI) fungicides, such as tebuconazole and prochloraz, are effective against *Fusarium* spp. [39]. Chitin is a linear polymer of *β*-(1,4)-linked *N*-acetylglucosamine (Glc-NAc) that forms the inner layer of the fungal cell wall forms during the morphogenesis. Chitinases catalyze the hydrolysis of cleavage of *β*-(1,4) glycosidic bonds between GlcNAc residues. Chitinase inhibitors, such as acetazolamide and bisdionin C, prevent phytopathogenic fungi growth and virulence [40]. *N*-Myristoyl transferase (NMT) catalyzes the transfer of the 14-carbon saturated fatty acid myristate from myristoyl-CoA to the *N*-terminal glycine residue through a variety of eukaryotic sources, including fungi, and, therefore, is an attractive target for the development of antifungal drugs or pesticides [41,42]. The plant cell wall consists of a network of complex carbohydrates, such as cellulose, hemicellulose, and pectin. As a cell wall-degrading enzyme, endoglucanase hydrolyzes polysaccharides during cell wall penetration into the fungal host. The endoglucanase I was found in *Macrophomina phaseolina* [6] and *Fusarium oxysporum* [43]. Phytopathogenic fungal proteases are important virulence factors during different aspects of the infection process, including adhesion to host cells, initial penetration of the plant cell wall, and colonization [44,45]. Proteinase K is a serine fungal protease that is involved in keratin degradation [46]. Pectine polygalacturonate is an important component of the cell wall and the middle lamella. Plant pathogens degrade cell wall through secreted polygalacturonases that hydrolyze cell wall during infection [47,48].

Recently, a paper was published that discussed the inhibitory effects of coumarin derivatives on the plant pathogenic fungi, as well as beneficial bacteria and nematodes [49]. New compounds were synthesized via principles of green chemistry and using deep eutectic solvents (DES) [50,51]. Coumarin derivatives were most effective against *Macrophomina phaseolina* and *Sclerotinia sclerotiorum*; however, they were not harmful against beneficial soil organisms, nematodes (*Heterorhabditis bacteriophora* and *Steinernema feltiae*), or the beneficial soil bacteria *Bacillus mycoides* and *Bradyrhizobium japonicum.* We obtained a predictive QSAR model for the antifungal activity against *M. phaseolina* and elucidated the binding mode with the active site of plant wall-degrading enzymes and proteinase K using the molecular docking method. We also studied the inhibitory effects of coumarinyl Schiff bases against the plant pathogenic fungi (*Fusarium oxysporum* f. sp. *lycopersici*, *Fusarium culmorum*, *M. phaseolina*, and *S. sclerotiourum*). The compounds were demonstrated to be efficient antifungal agents against *M*. *phaseolina* [52]. The results of molecular docking on the six enzymes related to the antifungal activity suggested that the tested compounds act against plant pathogenic fungi, inhibiting plant cell-wall-degrading enzymes, such as endoglucanase I and pectinase. Lethal for nematode, *H. bacteriophora* was proven to be associated with an inhibitory effect against acetylcholinesterase (AChE). The toxicity of observed compounds was estimated using T.E.S.T. v5.1. software.

The aim of this study was to evaluate the antifungal activities of the ten fluorinated 4,5-dihydro-1*H*-pyrazole derivatives [15] on the growth of the four phytopathogenic fungi (*F. oxysporum*, *F. culmorum*, *M. phaseolina* and *S. sclerotiorum*). Since the active components of plant protection products must be highly specific and environmentally and toxicologically acceptable, we tested their nematicidal on the following entomopathogenic nematodes: *Heterorhabditis bacteriophora* and *Steinernema feltiae*, which are beneficial soil organisms. Moreover, their environmental effects were tested on two soil-beneficial bacteria: *Bacillus mycoides* (gram-positive) and *Bradyrhizobium japonicum* (gram-negative). The toxicity and “pesticide-likeness” properties of observed compounds were estimated using in-silico methods. To determine the possible mechanism of action of pyrazole aldehydes against pathogenic fungi, a molecular docking study was carried out on three enzymes responsible for fungal growth—demethylase (sterol 14-demethylase (CYP51), chitinase, and transferase (*N*-myristoyltransferase)—and the three plant cell-wall-degrading enzymes—endoglucanase I, proteinase K, and pectinase (endopolygalacturonase).

Since acetylcholinesterase (AChE) is an enzyme responsible for the metabolism of acetylcholine (ACh), i.e., the neurotransmitter controlling motor activities in nematodes [53], we performed an AChE inhibition assay and compared it with the results of nematicidal activities. In order to determine the binding affinity and interactions between the most active compounds and AChE, molecular docking was performed.

This study reveals important information about the relationship between the structure and antifungal activities of fluorinated pyrazole aldehydes on four phytopathogenic fungi—*F. oxysporum*, *F. culmorum*, *M. phaseolina* and *S. sclerotiorum*—as well as their environmental effects. That knowledge will be used for the further development of pyrazoles as ecotoxicology-safe plant protection agents.

## 2. Results

### 2.1. Biological Activity Evaluation

#### 2.1.1. Antifungal Activity

The synthesis and characterization of fluorinated pyrazole aldehydes were reported previously [15]. Scheme 1 summarises the chemical structures of the analyzed compounds. The results of antifungal, antibacterial, and nematicidal assays are presented in Table 1. The tested compounds showed low activities against *M. phaseolina* and *F. oxysporum* f. sp. *lycopersici*; however, the highest inhibition against both species was observed with the same compound: 2-chlorophenyl derivative, **H9** (29.76 and 34.54%, respectively). For all tested compounds, except **H3** and **H4**, statistically significant differences were indicated between them and the *M. phaseolina* control treatment. Tested fluorinated pyrazole aldehydes were moderately active against *S. sclerotiorum* and *F. culmorum*. The most active compound against both fungi species was again shown to be **H9** (43.07 and 46.75%, respectively). Moderate activity against *S. sclerotiorum* also exhibited 2,5-dimethoxyphenil derivate, **H7** (42.23%), while against *F. culmorum*, moderate activity exhibited 2-methoxyphenil derivate, **H2** (38.62%). There is no statistically significant difference between **H3**, **H8**, and **H10** with PDA control in the test with *S. sclerotiorum*. In treatment with *F. oxysporum* f. sp., statistically significant differences were determined between all tested compounds and the untreated control, except for **H3** and **H5**. For all tested compounds, statistically significant differences were indicated between them and untreated PDA in the test with *F. culmorum*.

#### 2.1.2. Antibacterial Activity

Table 1 presents the minimum inhibitory concentrations of all tested compounds against the beneficial soil bacteria *Bacillus mycoides* and *Bradyrhizobium japonicum*. Neither compound showed an inhibitory effect on bacterial growth.

#### 2.1.3. Nematicidal Activity

Nematicidal activities of tested compounds (expressed as % of mortality) are presented in Table 1. Neither compound exhibited nematicidal activity against beneficial nematode species *H. bacteriophora*, except **H9** (18.75%), while compounds **H1**, **H3**, **H8**, and **H9** caused very low mortality (0.25–2.5%) against *S. feltiae*.

**Table 1:** Results of biological activities assayed for ten fluorinated pyrazole aldehydes: (^a^ inhibition rate (%) of phytopathogenic fungi, 48 h after inoculation at concentration 0.08 μmol/mL); ^b^ minimum inhibitory of concentration of beneficial soil bacteria (MIC/μg mL^−1^); ^c^ percentage corrected mortality (%) of entomopathogenic nematodes 72 h after inoculation at concentration 500 μg/mL; ^d^ acetylcholinesterase (AChE) inhibition assay performed via Ellman protocol using donepezil as a standard inhibitor. Enzyme inhibition absorbance was measured for 10 min at a wavelength of 412 nm.

Code of Molecule ^1^	Antifungal Activity ^a,2^				Antibacterial Activity ^b^		Nematicidal Activity ^c^		Inhibition of AChE/% ^d,3^
	*Macrophomina phaseolina*	*Sclerotinia* *sclerotiorum*	*Fusarium oxysporum* f. sp. *lycopersici*	*Fusarium* *culmorum*	*Bacillus* *mycoides*	*Bradyrhizobium* *japonicum*	*Heterorhabditis* *bacteriophora*	*Steinernema feltiae*	
**H1**	23.81 ± 14.78 *	17.74 ± 10.46 *	9.42 ± 1.57 *	23.37 ± 3.89 *	>512	>512	0.00 ± 0.00	1.25 ± 0.00	44.20 ± 0.00 *
**H2**	10.42 ± 1.72 *	14.36 ± 10.46 *	18.05 ± 4.05 *	38.62 ± 5.25 *	>512	>512	0.00 ± 0.00	0.00 ± 0.00	50.00 ± 5.18 *
**H3**	7.44 ± 2.98	5.91 ± 1.69	3.92 ± 4.44	28.46 ± 6.64 *	>512	>512	0.00 ± 0.00	1.25 ± 0.00	37.00 ± 4.94 *
**H4**	8.18 ± 2.85	36.32 ± 4.25 *	17.27 ± 6.47 *	26.42 ± 5.25 *	>512	>512	0.00 ± 0.00	0.00 ± 0.00	60.00 ± 0.00 *
**H5**	16.37 ± 5.70 *	39.70 ± 12.75 *	1.57 ± 6.69	31.50 ± 6.94 *	>512	>512	0.00 ± 0.00	0.00 ± 0.00	74.40 ± 0.00 *
**H6**	11.16 ± 4.46 *	28.72 ± 6.47 *	22.76 ± 4.44 *	28.46 ± 3.32 *	>512	>512	0.00 ± 0.00	0.00 ± 0.00	55.70 ± 0.02 *
**H7**	13.39 ± 7.87 *	42.23 ± 1.95 *	26.39 ± 1.57 *	29.47 ± 3.89 *	>512	>512	0.00 ± 0.00	0.00 ± 0.00	63.30 ± 6.00 *
**H8**	20.83 ± 5.43 *	10.98 ± 7.49	25.90 ± 9.24 *	33.54 ± 3.89 *	>512	>512	0.00 ± 0.00	2.50 ± 0.00	59.60 ± 0.00 *
**H9**	29.76 ± 9.41 *	43.07 ± 10.46 *	34.54 ± 10.37 *	46.75 ± 4.07 *	>512	>512	18.75 ± 0.00	0.25 ± 0.00	79.50 ± 0.00 *
**H10**	15.63 ± 7.83 *	13.51 ± 18.09	20.67 ± 3.95 *	25.41 ± 2.03 *	>512	>512	0.00 ± 0.00	0.00 ± 0.00	72.60 ± 5.88 *
Control ^4^	0 ± 0.00	0 ± 0.00	0 ± 0.00	0 ± 0.00	/	/	/	/	/
Donepezil ^5^	/	/	/	/	/	/	/	/	99.89 ± 0.01
LSD	10.54	14.01	8.66	6.84	/	/	/	/	4.51

^1^ Structures of molecules are presented in Scheme 1. ^2^ Average of four replications. Mean ± standard deviation (SD) determined via Fisher’s test. Mean values were compared with controls and considered significantly different when *p* ≤ 0.05 (*). ^3^ Concentration of compounds in final reaction mixture was 0.1 mM. Average of three replications ± SD. Mean values were compared with standard inhibitor and considered significantly different when *p* ≤ 0.05 (*). ^4^ Untreated potato dextrose agar. ^5^ Standard inhibitor of AChE.

#### 2.1.4. AChE Inhibition

The AChE inhibition assay of the tested compounds showed a moderate-to-high potency for inhibition (Table 1). The highest inhibition was observed for compound **H9** (79.50%), which corresponds with the results of the nematicidal inhibition assay where the same compound displayed the strongest activity, thus indicating the correlation between AChE inhibition and nematicidal activity. Strong inhibition was also found for compounds **H5** (74.40%) and **H10** (72.60%), while moderate inhibition was observed for compounds **H7** (63.30%), **H4** (60.00%), and **H8** (59.60%). For all tested compounds, statistically significant differences were indicated between them and Donepezil as the standard inhibitor.

### 2.2. In-Silico Analyses

#### 2.2.1. “Pesticide-Likeness” and Toxicity Parameters

Calculated “pesticide-likeness” molecular descriptors (molecular weight (MW), lipophilicity (MLOGP), the number of hydrogen-bond donors (HBD), the number of hydrogen-bond acceptors (HBA), the number of rotatable bonds (RB), and the number of aromatic bonds (AB) are presented in Table 2.

According to the pesticide-likeness rule [54], all compounds completely satisfy the pesticide-likeness criteria (MW ≤ 435 Da; MLOGP ≤ 6; HBA ≤ 6; HBD ≤ 2; RB ≤ 9; AB ≤ 17), except **H6**, which has 18 aromatic bonds.

Using the T.E.S.T. (Toxicity Estimation Software Tool) software [55,56], lethal doses of rats, aquatic toxicity, mutagenicity, and bioaccumulation are evaluated. T.E.S.T. estimated acute aquatic toxicity using quantitative structure–activity (QSAR) methodologies via the nearest-neighbour method. The results are presented in Table 3.

However, the highest toxicity was estimated for compound **H4** (464.23 mg/kg bw). Aquatic toxicity of the compounds is presented as the toxicity ciliate model organism *Tetrahymena pyriformis*, the fish fathead minnow (*Pimephales promelas*), and the bioaccumulation factor. All compounds have approximately equal estimated inhibition of growth of *Tetrahymena pyriformis*, though, once again, the most toxic is **H4** (pIGC_50_ 48-h = 4.58 mol/L). **H4** is also the compound with the highest toxicity toward fathead minnows (pLC_50_ 96-h = 8.24 mol/L). Bioaccumulation refers to the absorption of compounds into an organism from the natural environment. Since all compounds have a logBAF lower than three, their bioaccumulation is not significant [57]. Potentially mutagenic compounds, which could induce revertant colony growth of *Salmonella typhimurium*, are as follows: **H1**, **H2**, **H3**, **H**5, **H6**, **H8**, and **H9** [55]. Since the oral LD_50_ values for rats for all compounds are in the range 300–2000 mg/kg, they are characterized as “harmful if swallowed” according to the recommendation of the Organization for Economic Co-operation and Development (OECD) [58].

**Table 3:** Estimated toxicity for ten fluorinated pyrazole aldehydes (**H1**–**H10**). Toxicity was calculated based on the aim of program T.E.S.T. v.4.1 using nearest neighbour method [56].

Code of Molecule	Oral Rat LD_50_ (mg/kg bw) ^a^	*Tetrahymena pyriformis* pIGC_50_ 48-h (mol/L) ^b^	Fathead Minnow pLC_50_ 96-h (mol/L) ^c^	Mutagenicity Value (Result) ^d^	Bioaccumulation Factor (logBAF/L kg^−1^) ^e^
**H1**	977.34 (NN)	4.69 (NN)	5.78	0.70 (pos)	1.37
**H2**	977.34 (NN)	4.69 (NN)	5.82	0.67 (pos)	1.39
**H3**	937.90 (NN)	4.58 (NN)	5.37	0.57 (pos)	1.51
**H4**	464.23 (NN)	5.10 (NN)	8.24	0.37 (neg)	1.30
**H5**	977.34 (NN)	4.69 (NN)	5.80	0.69 (pos)	1.40
**H6**	931.38 (NN)	4.58 (NN)	5.26	0.64 (pos)	1.27
**H7**	1075.71 (NN)	4.69 (NN)	6.13	0.45 (neg)	1.42
**H8**	1137.40 (NN)	4.58 (NN)	5.89	0.63 (pos)	1.55
**H9**	991.78 (NN)	4.58 (NN)	5.90	0.63 (pos)	1.63
**H10**	1020.09 (NN)	4.58 (NN)	5.33	0.31 (neg)	1.34

^a^ mg of compound per bodyweight of rat required to kill half of a tested population; ^b^ negative logarithm (pIGC_50_) the concentration (mol/L) of compound in water that causes 50% growth inhibition to *Tetrahymena pyriformis* after 48 h; ^c^ negative logarithm (pLC_50_) of concentration (mol/L) of compound in water that kills half of fathead minnow (*Pimephales promelas*) in 96 h; ^d^ estimated mutagenicity of compound on *Salmonella typhimuriu*; ^e^ logarithmic value of bioaccumulation factor (logBAF as a ratio of concentration of compound in tissue of an aquatic organism to its concentration in water/liters per kilogram of tissue); NN—nearest neighbour method (predicted toxicity is estimated through taking an average of three chemicals in training set that is most similar to test chemical). Most toxic values are shaded.

#### 2.2.2. Molecular Docking

We performed a molecular docking study in order to elucidate the possible mechanism of action of fluorinated pyrazole aldehydes. For the docking study, we used six target enzymes related to antifungal activity, i.e., three enzymes responsible for fungal growth—demethylase (sterol 14α-demethylase (CYP51), pdb ID: 5eah) [38], chitinase (pdb ID: 4txe) [40], and transferase (*N*-myristoyltransferase, pdb ID: 2p6g) [41]—and three plant cell-wall-degrading enzymes—endoglucanase I (pdb ID: 2ovw) [43], proteinase K (pdb ID: 2pwb) [45], and pectinase (endopolygalacturonase, pdb ID: 1czf) [47]. A molecular docking study was also used to gain a deeper insight into the binding mode of fluorinated pyrazolines to AChE (pdb ID: 1eve) [59]. All docking scores, which are expressed as energy-based scoring functions (kcal mol^−1^), are presented in Table 4.

**Figure 1:**
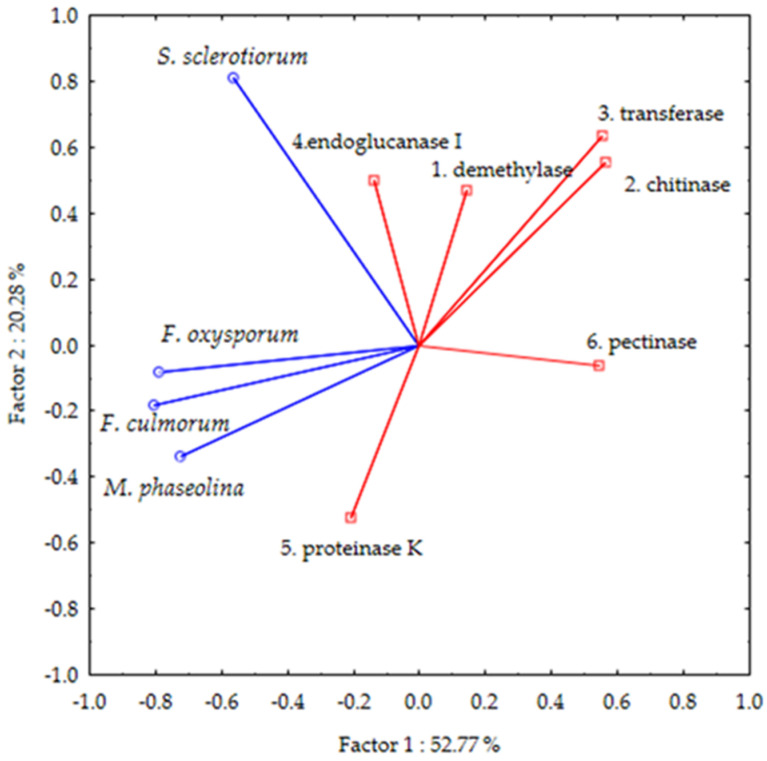
Projection of variables, antifungal activities of ten fluorinated pyrazole aldehydes against four plant pathogenic fungi (*Macrophomina phaseolina*, *Sclerotinia sclerotiorum*, *Fusarium oxysporum* f. sp. *lycopersici*, and *Fusarium culmorum*) (blue), and molecular docking scores energies (kcal mol^−1^) for six target enzymes related to antifungal activity (red) (three enzymes responsible for fungal growth: demethylase (sterol 14α-demethylase (CYP51), chitinase, and transferase (N-myristoyltransferase); three plant cell wall-degrading enzymes: endoglucanase, proteinase K, and pectinase (endopolygalacturonase)) on the factor-plane.

Principal components analysis (PCA) was performed in order to relate antifungal activities to the results of a molecular docking study obtained from six enzymes that were proven to be associated with antifungal activity. A biplot graph with the first two principal components for plant pathogenic fungi and six enzymes is presented in Figure 1. From the biplot, it is apparent that enzyme proteinase K, in which both factors of PCA have negative values, is placed in the same quadrant as three fungi: *M. phaseolina*, *F. culmorum*, and *F. oxysporum* f. sp*. lycopersic.* It implies that antifungal activity against these fungi is related to the inhibition of proteinase K. The inhibition of the growth of *S. sclerotiorum* is possibly related to the action of endoglucanase I since their variables are placed in the upper left quadrant. The other four enzymes are located in the opposite quadrant; thus, their activities could not be related to fungi growth inhibition. Since the strongest antifungal activity was obtained against *F. culmorum* (Table 1), further elucidation of the binding mode of the most effective inhibitor (compound **H2**) was performed on enzyme proteinase K. The same compound achieved the second strongest inhibition against *F. culmorum* (38.62%). The binding site was defined according to the crystal structure of the complex with coumarin (pdb ID: 2pwb). The energies of the main interactions between the binding site residues and **H2** in docked pose 1 are tabulated in Table 5.

**Figure 2:**
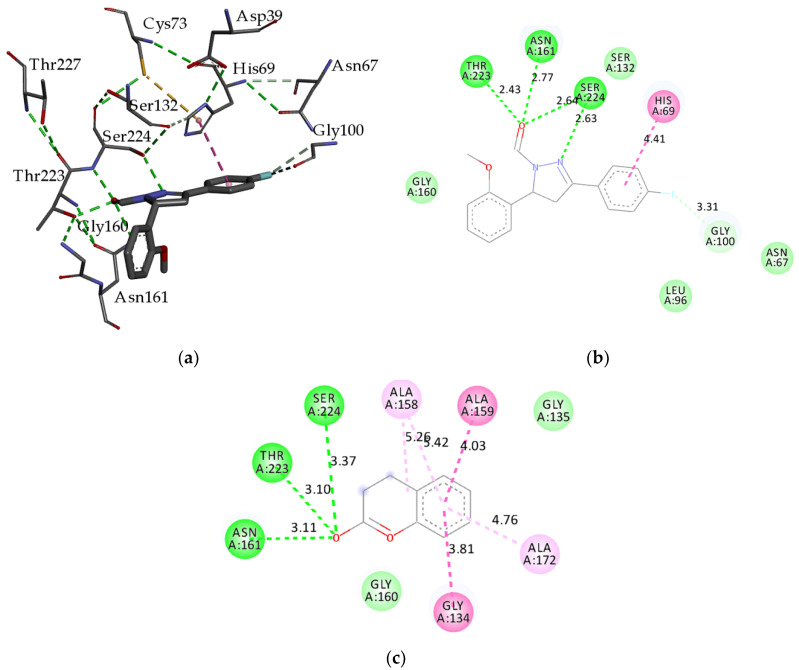
(**a**) Three-dimensional representation of interactions into binding site of proteinase K with docked fluorinated pyrazole aldehyde **H2**; two-dimensional diagram of main interactions and bond lengths (Å) with amino acid residues (**b**) of compound **H2**; (**c**) two-dimensional diagram of coumarin as standard ligand. (green = conventional hydrogen bond; light green = van der Waals; very light green = fluorine interactions; dark purple = π–π T-shaped interactions; pink = π-alkyl). Active site of proteinase K consists of catalytic triad Asp39-His69-Ser224. Receptor–ligand interactions were visualized with BIOVIA Discovery Studio Visualizer 4.5.

**Figure 3:**
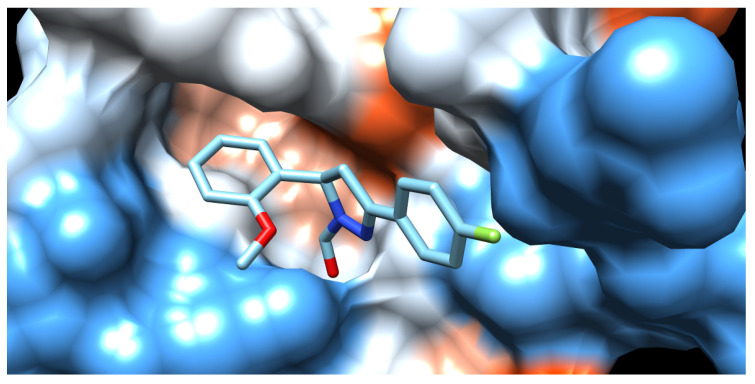
Hydrophobicity surface representation of proteinase K binding site with docked fluorinated pyrazole aldehyde **H2**. Dark blue for most hydrophilic amino acids, through white, to orange and red for most hydrophobic amino acids. Compound **H9** is located along active-site gorge, interacting with amino acid residues. Surface representation of enzyme binding site with docked compound was obtained using UCSF Chimera ver. 1.14.

The three-dimensional representation of the interactions of docked compound **H2** with amino acid residues of proteinase K is shown in Figure 2a. The two-dimensional diagrams of the main interactions of compounds, **H2**, and the standard inhibitor, coumarin, are presented in Figure 2b and Figure 2c, respectively. The hydrophobic surface representation of the proteinase K binding site with docked compound **H2** is presented in Figure 3.

Like most of the fungal proteolytic enzymes, proteinase K belongs to the serine proteases, which are named for the nucleophilic Ser residue at the active site (Asp-His-Ser) [44]. Thus, the active site of proteinase K consists of the catalytic triad Asp39-His69-Ser224, while the substrate recognition site is formed by pairs of residues, namely Gly100-Tyr104 and Ser132-Gly136 [60]. An oxygen atom from the carbonyl group of compound **H2** creates three H-bonds with Asn161 (2.77 Å), Ser224 (2.64 Å), and Thr223 (2.43 Å). The nitrogen atom from the pyrazole ring generates a second H-bond with Ser224 (2.63 Å). A very strong halogen bonding interaction is created between fluorine and Gly100 (3.31 Å). The side chain of His69 forms π–π T-shaped interactions with the phenyl ring.

The docking score reveals that some fluorinated pyrazole aldehydes are potentially better inhibitors of enzymes secreted by phytopathogen fungi than standard inhibitors. For example, the binding of all pyrazole aldehydes to proteinase K releases higher energy than coumarin. Such results could be explained based on the structure of fluorinated pyrazole aldehydes, which allows more and stronger interactions with the binding site residues. Coumarin also creates three hydrogen bonds between the 2-oxo group with Asn161, Ser224, and Thr223; however, since it is a smaller molecule, the lengths of these bonds are longer (3.11 Å; 3.37 Å; 3.10 Å, respectively) and, thereby, weaker (Figure 2c). The second hydrogen bond with Ser224 is omitted in coumarin because nitrogen atoms are absent from its structure, as well as fluorine interaction with Gly100.

In order to elucidate a binding mode of the fluorinated pyrazole aldehydes on the AChE enzyme responsible for the nematicidal activity, we performed molecular docking. The binding site was determined according to the complex of *N*-benzylpiperidine-based inhibitor or donepezil (E2020, pdb id: e20) with *Torpedo californica* AChE (pdb ID: 1eve). The ranking of the screened compounds with docking score energies is presented in Table 4. The highest binding energy was achieved usinh standard ligand E2020 (−117.34 kcal mol^−1^), followed by compounds **H4** (−108.34 kcal mol^−1^) and **H1** (−100.08 kcal mol^−1^), which exhibited moderate inhibition against AChE (60.00%, and 44.20%, respectively) (Table 1). Compound **H9**, which exhibited the highest nematicidal activity against *H. bacteriophora* (18.75%) and was the strongest inhibitor of AChE in vitro (79.50%) (Table 1), reached the binding energy of −95.08 kcal mol^−1^. The main interactions between compound **H9** and the binding energies with residues of AChE are presented in Table 6.

**Figure 4:**
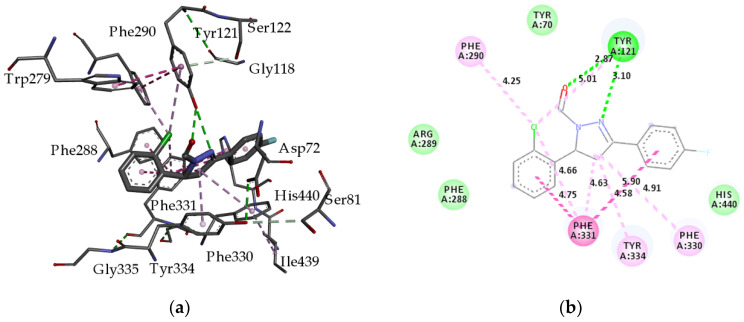
(**a**) Three-dimensional representation of interactions between fluorinated pyrazole aldehyde **H9** and amino acid residues of acetylcholinesterase (AChE); (**b**) two-dimensional diagram of main interactions and bond lengths (Å) with amino acid residues. (Green = conventional hydrogen bond; light green = van der Waals; dark purple = π–π T-shaped interactions; purple = π–π stacked interactions; light purple = π-alkyl interactions). Receptor-ligand interactions were visualized with BIOVIA Discovery Studio Visualizer 4.5.

**Figure 5:**
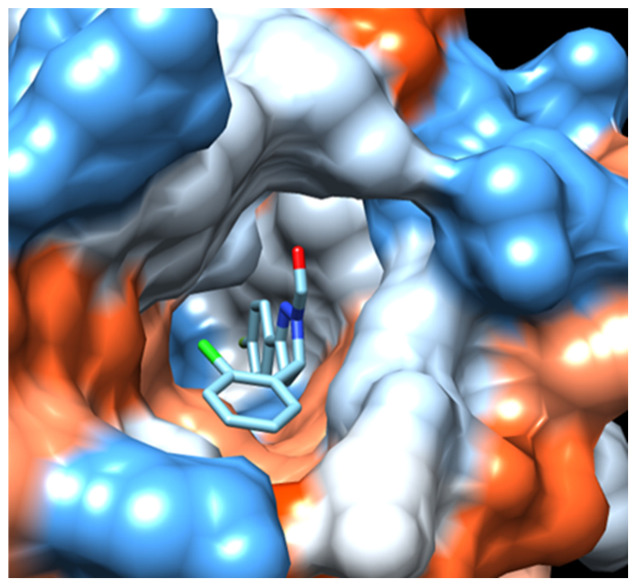
Hydrophobicity surface representation of acetylcholinesterase (AChE) active-site gorge with docked fluorinated pyrazole aldehyde **H9**. Dark blue for most hydrophilic amino acids, through white, to orange and red for most hydrophobic amino acids. Compound **H9** is located in peripheral active site (PAS), aromatic site (AS), acyl pocket (AP), and oxyanion hole (OH) of AChE, blocking narrow passage to catalytic site. Surface representation of enzyme binding site with docked compound was obtained using UCSF Chimera ver. 1.14.

Figure 4a,b show three- and two-dimensional representations, respectively, of interactions between compound **H9** and amino acid residues of AChE. The hydrophobicity surface representation of the acetylcholinesterase (AChE) active-site gorge with docked fluorinated pyrazole aldehyde **H9** is presented in Figure 5. Unlike ligand E2020 in the original complex (pdb ID: 1eve), which does not interact with the catalytic triade (Ser200, His440, and Glu327) at the catalytic active site (CAS) [61], compound **H9** interacts through fluorine with His440 via Van der Waals interaction. Compound **H9** is located along the active-site gorge (Figure 5), interacting with amino acid residues located in the peripheral active site (PAS, Tyr70, Tyr121, Trp279, and Tyr334); aromatic site; formerly called the anionic site (AS, Phe330, and Phe331); acyl pocket (AP, Phe288, and Phe290); and oxyanion hole (OH) of AChE, thus blocking the narrow passage to the catalytic site [61]. As seen in Figure 4, **H9** makes two hydrogen bond interactions with Tyr121 through its nitrogen atom from the pyrazole ring and oxygen atom from the carbonyl group. Two phenyl groups of **H9** create π–π T-shaped interactions with Phe331. Moreover, π-alkyl interactions that generate between the pyrazole ring and Phe330, Phe331, and Tyr334 are very relevant. The chlorine atom creates π-alkyl interactions with Tyr121, Phe290, and Phe331. The phenyl ring of compound **H9** also creates π–π stacked interactions with Phe331 in the anionic site of AChE.

## 3. Discussion

Fluorinated pyrazole aldehydes demonstrate low-to-moderate antifungal activities against phytopathogenic fungi (*M. phaseolina* and *F. oxysporum* f. sp. *lycopersici*). Among the tested derivative, **H9** stood out as the most potent. The same compound was shown to be safe against beneficial soil bacteria, though it demonstrated moderate inhibition activity against the beneficial nematode *H. bacteriophora.* Compound **H9** satisfies pesticide-likeness criteria and was evaluated as non-toxic for rats and aquatic organisms; however, it was assessed as potentially mutagenic. Although it is common knowledge that the pyrazole ring is a highly efficient pharmacophore for pesticide design, the results confirm previous findings that polyhalogenated phenylpyrazole rings may yield more potent pesticides [62]. Moreover, difluoromethylpyrazole acyl urea derivatives were identified as promising compounds for the development of new fungicides, especially against *Botrytis cinerea* [63]. Compounds containing an active 3-trifluoromethyl-1*H*-pyrazole-4-carboxamide skeleton displayed better activities against phytopathogenic fungi than those of the commercial fungicides carboxin and boscalid [64]. Contrary to the above results, the study by Sun and Zhou [25] showed that involving trifluoro-methyl instead of methyl at the C-3 of the pyrazole ring of isoxazolol pyrazole carboxylate significantly weakened antifungal activity against four types of phytopathogenic fungi. Thiophene pyrazole derivatives containing a benzothiazole moiety showed 50% lower inhibitory activity against *F. oxysporum* than the standard fungicide, i.e., cycloheximide [65]. The results revealed that additional nitrogen and sulfur atoms in pyrazole derivatives enhance antifungal activity Based on the above results, future fluorinated pyrazole aldehydes should include benzothiazole and thiophene moiety to enhance their antifungal effects.

During the penetration and colonization of the host plant, phytopathogenic fungi secrete various proteolytic enzymes that are involved in the degradation of the host’s extracellular matrix during an invasion. Fungal proteases are important during infection, including adhesion to host cells, initial penetration of the plant cell wall, and colonization. Since proteases increase the permeability of the plant plasma membrane, their activities are strongly related to disease aggressiveness in several plant pathogenic fungi. [44,61,66]. One of the most active proteases is serine proteinase K, which hydrolyzes the peptide bonds via a nucleophilic serine residue at the active site [60]. A study by Pekkarinen et al. [67] proved that proteinase activity was related to *F. culmorum* growth, which supports our docking study results. Therefore, proteinase K is a suitable target for the elucidation of the structure–activity relationship of its inhibitors. Two serine protease inhibitors contain fluorine as substituents: organophosphorus nerve agent diisopropylfluorophosphate (DFP) and phenylmethylsulfonyl fluoride (PMSF) [45]. Coumarins demonstrated high inhibitory potency against various serine proteases, especially halomethyl dihydrocoumarin and isocoumarins [68]. Hydrolytic enzymes, such as cellobiohydrolases and endoglucanases, are plant cell-wall-degrading enzymes that enable cellulose breakdown and penetration into host tissue by fungal species. Endoglucanase I enzyme catalyze the hydrolysis of the *β*-1,4-glycosidic linkages of cellulose. The active sites of endoglucanase I are enclosed by a long open canyon formed by the concave curvature of the “upper” *β*-sheet. The binding groove is located deep at the bottom of this canyon and plays a key role in catalysis [36]. It was isolated from *Fusarium oxysporum* [43,69] and *Macrophomina phaseolina* [6,70]. A unique pathogen-specific endoglucanase (*egl1*) gene was found in *M. phaseolina*. Its nucleotide sequence reveals that *M. phaseolina egl1* lacks a cellulose binding domain, which is a universal feature in saprophyte endoglucanases [71]. Recently, we demonstrated that coumarin derivatives act as efficient antifungal agents against *M. phaseolina* and *S. sclerotiorum*. The results of molecular docking suggested that these compounds probably inhibit cell-wall-degrading enzymes, such as endoglucanase I and pectinase [49,52]. Molecular docking studies confirm the previous conclusion that additional nitrogen atoms in the structure of pyrazole could produce more hydrogen bonds with residual binding sites, enhancing their inhibitory activity.

The environmental effects of tested pyrazoles were satisfactory. Compounds did not demonstrate harmful effects on beneficial soil gram-positive and gram-negative bacteria or on entomopathogenic nematodes. Beneficial soil plays a major role in maintaining the fertility and health of the soil, and the actions of newly developed plant protection agents, such as fluorinated pyrazole aldehydes, which work strongly in suppressing the fungal causative agents of plant diseases and do not impair the dynamics and abundance of the soil bacteria beneficial population, are extremely valuable. Similar results were obtained from the research of Guo et al. [29], who evaluated novel *N*-phenylpyrazole fraxinellone hybrid compounds and, in general, observed less potent activity than in the control. One compound showed potent antibacterial activity against the pathogen gram-positive bacterial strain *Bacillus subtilis* with a recorded MIC value of 4 mg mL^−1^. In the research of Rastija et al. [15], the inhibitory effect of fluorinated pyrazoles on pathogen gram-positive and gram-negative bacteria was determined, and increased resistance was observed in gram-positive bacteria with recorded MIC values from 62.5–250 µg mL^−1^ for *Bacillus subtilis* and *Staphylococcus aureus*. In the research of Kumar et al. [20], a novel sequence of pyrazole derivatives was synthesized and some derivatives exhibited high activity against (MIC: 0.25 µg mL^−1^) gram-negative *Escherichia coli* and *Streptococcus epidermidis*. Entomopathogenic nematodes (EPNs) are beneficial organisms used in pest management programs against insect pests. Further development and discovery of new plant protection compounds should be safe for non-target organisms, such as EPNs. Most of the tested compounds in this study proved to be safe for EPNs and were species-specific. Only compound **H9** caused mortality of more than 10% in the population of EPNs, and this result was recorded only against *H. bacteriophora.* Novel pyrazole and fluorinated pyrazole carboxamides were shown to possess strong nematocidal properties with an inhibition rate of at least 80% at a dose of 40 mg/L against plant parasitic nematodes in tomato seedlings, which is contrary to our findings [72,73]. *S. feltiae* proved to be more resistant to tested compounds compared to *H. bacteriophora,* indicating differences in metabolism between species [74].

As we expected, the nematicidal activity of compound **H9** is in accordance with the inhibition effect of AChE. To understand the binding modes of tested pyrazoles and their interactions with AChE, we performed a molecular docking study according to the structure of AChE complexed with donepezil acting as an inhibitor (pdb ID: 1eve). The most relevant sites of AChE for ligand binding are the catalytic active site (CAS) and the peripheral aromatic site (PAS), while the W-loop (OML), acyl pocket (AP), and oxyanion hole (OH) sites are essential in contributing to binding affinity and complex stability [75]. It was demonstrated that donepezil-pyridyl hybrids inhibit both cholinesterases at the PAS and CAS regions, suggesting that the *N*-alkyl bridge enhances AChE inhibition using such pyridine-based derivatives [76]. Halogenated derivatives of donepezil, especially CF_3_ derivative, showed strong AChE inhibiting potential, and molecular docking indicated that these compounds have non-covalent interactions with hydrophobic gorges and aromatic subsites of AChE, which is in accordance with our results (Figure 5) [77]. A series of fluorined pyrazole carboxamides showed powerful nematocidal activity against *Meloidogyne incognita*. Compounds with electron-donating groups located at 4- or 2,6-positions (-*t*-Bu; -*O*-Phe; -Et; -CH_3_) of the benzene ring exhibited significant nematicidal activity. Docking results on AChE showed similar results to those of our study. The best active compounds interact with amino acid residue Tyr 121 and Trp 279 of AchE via hydrogen bonding [73]. A series of chromone derivatives containing substituted pyrazole exhibited high nematicidal activity in vivo against *M. incognita*. A molecular docking study performed on the AChE indicated that one of the most active compounds generated H-bond with the OH group of Ty121, as well as the *π*–*π* stacking interaction with the Phe330 and Trp84 residue [78]. On the contrary, two neuroactive insecticides chlorpyrifos and pirimicarb, which are inhibitors of AchE enzyme, did not affect nematode *Steinernema feltiae* survival [79].

AChE has a critical role in terminating nerve impulses in the cholinergic nervous system of most animals, catalyzing the hydrolysis of the neurotransmitter acetylcholine (ACh) to acetic acid and choline. Inhibition of AChE activity leads to a reduction in the hydrolysis and accumulation of acetylcholine in the synaptic cleft, hyperstimulation of nicotinic and muscarinic receptors, disruption of neurotransmission, and, finally, death [80]. Therefore, inhibitors of AChE are active components of pesticides, the toxic effect of which is associated with AChE inhibition [53]. The organophosphorus compounds (OPs) and carbamate compounds are used in agriculture as insecticides. OPs phosphorylate serine residues of AChE in a non-reversible way. Carbamates reversibly inhibit AChE since the AChE-carbamate complex is unstable and the carbamyl moiety can be split from the enzyme via spontaneous hydrolysis [81].

In our previous study, the relationship between nematicidal activity and inhibitory effect against AChE of coumarinyl Schiff bases was also confirmed [52]. The study of Singh et al. (2017) [82] showed that AChE activity is drastically inhibited in insects exposed to the natural insecticide 2,3-dimethylmaleic anhydride. Since plant essential oils and their constituents showed activity against pinewood nematode, i.e., *Bursaphelenchus xylophilus*, the inhibitory activity of several phytochemicals derived from plant essential oils against AChEs of *B. xylophilus* (BxACEs) was evaluated. Inhibition of *B. xylophilus* was directly related to inhibition of its BxACEs, suggesting the possibility of using anti-BxACE activity assays for screening and developing novel nematicides [53].

Besides the derivative with styryl group (Ph-CH=CH-R), toxicity to aquatic organisms and rat was not calculated for other compounds. Although the styryl group is widely represented in medicinally important compounds, *α*,*β*-unsaturated carbonyl group (Michael acceptor site) in the styryl group makes it amenable to transformation into toxic metabolites [83]. According the calculated bioaccumulation factor, the pyrazoles should not accumulate in the environment, which makes them safe to use. Overall, the pyrazoles showed a low environmental impact. In a further study of fluorinated pyrazole aldehydes, great attention should be paid to their mutagenicity. In designing new compounds, it is necessary to omit structural moieties that affect mutagenicity.

## 4. Materials and Methods

### 4.1. Biological Assays

Structures of fluorinated pyrazole aldehydes are presented in Scheme 1. Their names are as follows: 3-(4-fluorophenyl)-5-(4-methoxyphenyl)-4,5-dihydro-1*H*-pyrazole-1-carbaldehyde (**H1**); 3-(4-fluorophenyl)-5-(2-methoxyphenyl)-4,5-dihydro-1*H*-pyrazole-1-carbaldehyde (**H2**); 5-(3-fluorophenyl)-3-(4-fluorophenyl)-4,5-dihydro-1*H*-pyrazole-1-carbaldehyde) (**H3**) (*E*)-3-(4-fluorophenyl)-5-(4-styrylphenyl)-4,5-dihydro-1*H*-pyrazole-1-carbaldehyde (**H4**); 3-(4-fluorophenyl)-5-(3-methoxyphenyl)-4,5-dihydro-1*H*-pyrazole-1-carbaldehyde (**H5**); 3-(4-fluorophenyl)-5-(2-hydroxyphenyl)-4,5-dihydro-1*H*-pyrazole-1-carbaldehyde (**H6**); 5-(2,5-dimethoxyphenyl)-3-(4-fluorophenyl)-4,5-dihydro-1*H*-pyrazole-1-carbaldehyde (**H7**); 5-(4-bromophenyl)-3-(4-fluorophenyl)-4,5-dihydro-1*H*-pyrazole-1-carbaldehyde (**H8**); 5-(2-chlorophenyl)-3-(4-fluorophenyl)-4,5-dihydro-1*H*-pyrazole-1-carbaldehyde (**H9**); and 5-(4-(dimethylamino)phenyl)-3-(4-fluorophenyl)-4,5-dihydro-1H-pyrazole-1-carbaldehyde (**H10**).

#### 4.1.1. Antifungal Assays

Antifungal assay was performed in vitro using nine pyrazole aldehydes against phytopathogenic fungi *F. oxysporum* f. sp. *Lycopersici*, *F. culmorum*, *M. phaseolina*, and *S. sclerotiourum*. Fungal cultures used in this research were provided from the culture collections of the Chair of Phytopathology, Faculty of Agrobiotechnical Sciences, Osijek, Croatia. For the preparation of stock solutions of compounds, a concentration of 4 μmol mL^−1^ corresponding mass was dissolved in 2.5 mL of dimethyl sulfoxide (DMSO, Merck, Darmstadt, Germany) and 2.5 mL of distilled water. The volume of 1 mL of stock solution was added to the mixture to produce the final compound concentration of 0.08 μmol mL^−1^, as well as to keep the amount of DMSO in the mixture at 1%. The antifungal test was performed using the methodology described by Siber et al. [84]. The Petri dishes (9 cm in diameter) were filled with a mixture of potato dextrose agar (PDA) (Biolife, Milan, Italy) and one type of selected pyrazole aldehydes compound. Agar plugs (4 mm diameter) of one-week-old culture were picked up with a sterilized needle and placed in the center of the Petri dishes. Petri dishes were maintained in a growth chamber at 22 ± 1 °C, with a 12 h light/12 h dark regime. Each treatment was performed in four replicates. As a control, untreated potato dextrose agar was used. The diameter of the aerial mycelium was measured 48 h after inoculation, and the influence of the examined compounds on the fungi was calculated using the antifungal index (% inhibition) [85].

#### 4.1.2. Antibacterial Assays

Antibacterial activity was assessed using the brot microdilution assay, as described in Wiegand et al. [86], with the aim of determining the minimum inhibitory concentration (MIC) of fluorinated pyrazole aldehydes. Tested micro-organisms were beneficial soil gram-positive (*Bacillus mycoides)* and gram-negative (*Bradyrhizobium japonicum*) bacteria from the bacterial collection of the Faculty of Agrobiotechnical Sciences, Osijek, Croatia. Pure bacterial cultures were obtained via cultivation on nutrient (Liofilchem, Roseto degli Abruzzi, Italy) and Vincent agar (mannitol 10 g, K_2_HPO_4_ 0.5 g, MgSO_4_ × 7H_2_0 0.2 g, NaCl 0.1 g, yeast 0.5 g). Pyrazole aldehydes were dissolved in 20 μL of DMSO with the addition of sterile water up to 200 μL. The compounds were diluted from 512 to 1 μg mL^−1^ in a sterile 96-vell plate. Bacterial suspension is adjusted to 0.5 McFarland standard scale and diluted to 1.5 × 10^5^ cells (CFU mL^−1^). Each well was inoculated with 50 µL of pure bacterial isolate. Growth and sterile control were included in each plate. After incubation, the MIC of the compound was determined in wells without visual turbidity in the four-repetition assay.

#### 4.1.3. Nematicidal Assays

Before the nematicidal assay, the infective juveniles (IJ) were maintained at 4 °C at a density of 2000 IJ/mL. For the analysis, we used only IJ that were less than 2 weeks old [87]. Nematode mortality was checked for all compounds at a maximum concentration of 500 μg/mL with four repetitions in a 24-well plate under stereo zoom microscope Olympus SZX16 (Tokyo, Japan). Each compound was dissolved in DMSO and distilled water containing 0.1% Triton to create the stock solution. The control treatment was conducted with distilled water containing DMSO and Triton. Nematode mortality was monitored over a 48-hour period. Approximately 100 infective juveniles of *H. bacteriophora* (Croatian strain ISO9, Gen-Bank accession numbers MG944244) and *S. feltiae* (Croatian strain ISO16, GenBank accession numbers MG952287) were placed separately in each well containing 250 μL of the working solution. Well plates were maintained in the dark at 24 °C. Mortality was detected when nematodes failed to respond to physical stimuli with a probe. For mortality correction, the Schneider–Orelli formula was used.

#### 4.1.4. Acetylcholinesterase Inhibition Assays

AChE (EC 3.1.1.7, type VI-S from *Electrophorus electricus* (electric eel)), donepezil, and bovine serum albumin (BSA) were purchased from Sigma–Aldrich (St. Louis, MO, USA). Dimethyl sulfoxide (DMSO) was purchased from Merck (Darmstadt, Germany). Acetylthiocholine iodide (ATChI) was purchased from Alfa Aesar (Haverhill, MA, USA). 5,5′-Dithiobis(2-nitrobenzoic acid) (DTNB) was purchased from VWR International (Radnor, PA, USA). All other chemicals used were of analytical grade. Acetylcholinesterase inhibition assay was performed via Ellman protocol using donepezil as a standard inhibitor [88]. Compounds were dissolved in DMSO and phosphate buffer solution (pH 8.0); thus, the final reaction mixture contained 0.1 mM concentration of tested compounds and less than 1% DMSO. For preparation, the enzyme stock solution AChE was dissolved in a phosphate buffer solution (pH 8.0) containing 0.1% (*w*/*v*) BSA. The reaction mixture contained 10 µL of test compound solution, 130 µL of phosphate buffer, 20 µL of AChE solution (0.25 U/mL), 20 µL of DTNB solution, and 20 µL of ATChI solution (15 mM).

The mixture was incubated for 15 min at 25 °C, and a substrate (ATChI) was added. Enzyme inhibition absorbance was measured for 10 min at a wavelength of 412 nm using a microplate reader (iMark, Bio-Rad, Hercules, CA, USA). Inhibition activity was expressed as % inhibition:

Inhibition % = *A*_0_ − *A*_1_/*A*_0_ × 100 (1)

where *A*_1_ is the absorbance of the tested compound, and *A*_0_ is the absorbance of the positive control.

### 4.2. Computational Methods

#### 4.2.1. Calculation of Pesticide-Likeness Molecular Descriptors

Molecular weight (MW), lipophilicity (MLOGP), the number of hydrogen-bond donors (HBD), the number of hydrogen-bond acceptors (HBA), the number of rotatable bonds (RB), and the a number of aromatic bonds (AB) of tested compounds were calculated using ADMEWORKS ModelBuilder (Version 7.9.1.02011 Fujitsu Kyushu Systems Limited, Krakow, Poland).

#### 4.2.2. Calculation of Toxicity

The toxicity of compounds was calculated via the aim of program T.E.S.T. v.4.1 (US EPA Research, Durham, NC, USA) using the nearest neighbour method [56]. Estimated toxicity was as follows: lethal dose for rats (oral rat LD_50_/mg/kg of body weight), aquatic toxicity against *Tetrahymena pyriformis* (48-h *T. pyriformis* IGC_50_) [89], and 96-h fathead minnow (c/mol L^−1^) [35]. The estimated Ames mutagenicity of the compound was expressed as the result of the bacterial reverse mutation assay performed on *Salmonella typhimurium* [90]. The bioaccumulation factor (BAF) was expressed as a logarithmic value of the ratio of the concentration of a compound in the fish and the water (L/kg tissue) [57].

#### 4.2.3. Molecular Docking

Prior to the molecular docking study, the structures of the compounds were optimized via Spartan ’08 v1.2.0. (Wavefunction, Inc.; Irvine, CA, USA, 2009) using the molecular mechanics force field (MM+) [91] and, subsequently, via the semiempirical AM1 method [92].

For the elucidation of the mechanism of fungal pathogenesis, we performed molecular docking studies of tested compounds on six enzymes responsible for these activities (pdb ID: 5eah, 4txe, 2p6g, 2ovw, 2pwb, and 1czf). For the molecular docking study of tested compounds on AChE, we used AChE with donepezil as an inhibitor (pdb ID: 1eve). Crystal coordinates of enzymes in complex with docked ligands were extracted from Protein Data Bank (PDB, https://www.rcsb.org/) (accessed on 15 November 2022). The molecular docking of compounds was performed using iGEMDOCK (BioXGEM, Hsinchu, Taiwan). Compounds were docked via a generic evolutionary method using the following parameters: population size: 200; generations: 70; and the number of poses: 3. iGEMDOCK generated protein compound complexes and interaction profiles (based on electrostatic (E), hydrogen-bonding (H), van der Waals (V), and electrostatic interactions (Elec). Finally, compounds were ranked based on their total binding energy using an energy-based scoring function: Total Energy (kcal/mol) = vdW + Hbond + Elec [93]. Receptor–ligand interactions were visualized with BIOVIA Discovery Studio Visualizer 4.5 (Dassault Systèmes, San Diego, CA, USA), while a surface representation of the enzyme binding site with the docked compound was performed using UCSF Chimera ver. 1.14 (University of California, San Francisco, CA, USA).

#### 4.2.4. Statistical Analysis

ANOVA was performed with the aim of SAS/STAT. User’s guide, version 9.2, SAS Institute Inc., Cary, NC, USA. 2008. Principal component analysis was performed with Statistica 14 (TIBCO Software Inc. 2020, Palo Alto, Santa Clara, CA, USA).

## 5. Conclusions

The fluorinated pyrazole aldehydes analysed in this study showed moderate antifungal properties against phytopathogenic fungi, as well as environmental, toxicological, and pesticide-likeness acceptable properties. These compounds were shown to have the potential for further development as future plant protection products.

## Figures and Tables

**Table 2:** **“**Pesticide-likeness” molecular descriptors for ten fluorinated pyrazole aldehydes (**H1**–**H10**) calculated using ADMEWORKS ModelBuilder tool.

Code of Molecule	MW	MLOGP	HBA	HBD	RB	AB
**H1**	298.34	3.211	5	0	3	12
**H2**	298.34	3.211	5	0	3	12
**H3**	286.3	3.899	5	0	2	12
**H4**	370.45	5.007	4	0	4	18
**H5**	298.34	3.211	5	0	3	12
**H6**	284.31	2.971	5	1	2	12
**H7**	328.37	2.926	6	0	4	12
**H8**	347.2	4.137	4	0	2	12
**H9**	302.75	4.019	4	0	2	12
**H10**	311.39	3.446	5	0	3	12

MW (molecular weight); MLOGP (Moriguchi’s octanol–water partition coefficient); HBA (number of hydrogen bond acceptors); HBD (number of hydrogen bond donors); RB (number of rotatable bonds); AB (number of aromatic bonds). Rule-violating parameter is shaded.

**Table 4:** Docking score energies (Total E/kcal mol^−1^) of docked poses of ten pyrazole aldehydes (**H1**–**H10**), including standard inhibitor in complex with demethylase (sterol 14α-demethylase (CYP51), pdb ID: 5eah), chitinase (pdb ID: 4txe), transferase (N-myristoyltransferase, pdb ID: 2p6g), endoglucanase I (pdb ID: 2ovw), proteinase K (pdb ID: 2pwb), pectinase (endopolygalacturonase, pdb ID: 1czf), and AChE (acetylcholinesterase, pdb ID: 1eve). Molecular docking of compounds was performed using iGEMDOCK via a generic evolutionary method and using the following parameters: population size: 200; generations: 70; number of poses: 3.

Demethylase		Chitinase		Transferase		Endoglucanase I		Proteinase K		Pectinase		AChE	
Code of Mol.	Total E	Code of Mol.	Total E	Code of Mol.	Total E	Code of Mol.	Total E	Code of Mol.	Total E	Code of Mol.	Total E	Code of Mol.	Total E
**H4**	−85.20	38f *	−120.51	3lp *	−87.65	**H4**	−106.52	**H2**	−97.70	**H10**	−76.66	e20 *	−117.34
**H1**	−81.74	**H4**	−104.99	**H4**	−82.74	**H5**	−103.78	**H10**	−94.29	**H1**	−75.23	**H4**	−108.34
5lw *	−80.98	**H6**	−98.86	**H6**	−81.44	in1 *	−101.69	**H9**	−92.93	**H4**	−74.98	**H1**	−100.08
**H7**	−79.88	**H5**	−97.41	**H5**	−77.38	**H6**	−101.60	**H5**	−87.31	**H6**	−73.74	**H10**	−99.86
**H2**	−78.71	**H1**	−96.77	**H1**	−76.96	**H2**	−101.03	**H8**	−86.93	Nag *	−73.49	**H5**	−95.48
**H5**	−78.21	**H3**	−96.74	**H7**	−76.42	**H1**	−99.22	**H6**	−86.36	**H3**	−73.31	**H9**	−95.08
**H8**	−77.94	**H7**	−96.31	**H3**	−75.19	**H9**	−96.67	**H3**	−86.33	**H8**	−73.26	**H7**	−94.89
**H6**	−77.39	**H10**	−95.94	**H10**	−73.74	**H8**	−92.21	**H4**	−81.16	**H5**	−72.97	**H6**	−93.44
**H10**	−76.52	**H8**	−94.86	**H8**	−73.14	**H7**	−91.48	**H1**	−81.08	**H7**	−71.24	**H2**	−93.09
**H9**	−76.42	**H2**	−89.52	**H2**	−72.15	**H10**	−90.50	**H7**	−76.08	**H9**	−70.82	**H3**	−92.24
**H3**	−75.40	**H9**	−88.97	**H9**	−68.58	**H3**	−83.88	Cou *	−72.31	**H2**	−70.26	**H8**	−90.52

* pdb ID of standard ligands: 1-[[(2~{S},4~{S})-2-[2-chloranyl-4-(4-chloranylphenoxy)phenyl]-4-methyl-1,3-dioxolan-2-yl]methyl]-1,2,4-triazole (**5lw**); (2S)-1-(2,3-dihydro-1H-inden-2-ylamino)-3-(3,4-dimethylphenoxy)propan-2-ol (**38f**); (2S)-1-(cyclohexylamino)-3-(6-methyl-1,2,3,4-tetrahydrocarbazol-9-yl)propan-2-ol (**3lp**); 4-(beta-D-glucopyranosyloxy)-2,2-dihydroxybutyl propanoate (**in1**); coumarin (**cou**); 2-acetamido-2-deoxy-beta-D-glucopyranose (**nag**); (2R)-5,6-dimethoxy-2-[[1-(phenylmethyl)piperidin-4-yl]methyl]-2,3-dihydroinden-1-one (**e20**).

**Table 5:** Energies (kcal mol^−1^) of main interactions between fluorinated pyrazole aldehyde **H2** and binding site residues of proteinase K (a serine fungal protease that is involved in fungal cell wall degradation) (pdb ID: 2pwb).

Residue	Energy (kcal mol^−1^)	Residue	Energy (kcal mol^−1^)
Hydrogen bond		Van der Waals interactions	
S-Asn161	−6.68	M-Ala159	−2.35
M-Thr223	−2.76	M-Gly160	−7.12
S-Thr223	−1.10	M-Asn161	−4.47
M-Ser224	−3.50	S-Asn161	−3.05
S-Ser224	−9.50	S-Asn162	−3.02
Van der Waals interactions		S-Tyr169	-3.46
S-Asn67	−0.85	M-Thr223	−1.60
S-Leu96	−0.69	M-Ser224	−1.93
M-Asn99	−1.14	S-Ser224	−3.55
M-Ser132	−5.26	Fluorine interactions	
M-Leu133	−8.13	M-Gly100	−1.32
M-Gly134	−6.89		
M-Gly135	−3.01	π–π T−shaped interactions	
M-Ala158	−2.11	S-His69	−8.06

M = main chain; S = side chain.

**Table 6:** Energies (kcal mol^−1^) of main interactions between fluorinated pyrazole aldehyde **H9** and residues of acetylcholinesterase (AChE, pdb ID: 1eve). AChE is an enzyme responsible for metabolism of acetylcholine (ACh), which is neurotransmitter controlling motor activities in nematodes.

Residue	Energy (kcal mol^−1^)
hydrogen bond	
S-Tyr121	−7.07
S-Tyr121	−6.07
Van der Waals interactions	
S-Tyr70	−1.31
M-Trp84	−0.02
M-Gly117	−0.45
S-His440	−3.87
S-Trp279	−1.09
π–π stacked interactions	
S-Phe290	−1.09
π–π T-shaped interactions	
M-Phe331	−5.43
π-alkyl interactions	
S-Phe330	−13.08
M-Phe331	−15.11
S-Tyr334	−10.83

M = main chain; S = side chain.

## Data Availability

Data are presented within the article.
